# Altering calcium influx for selective destruction of breast tumor

**DOI:** 10.1186/s12885-017-3168-x

**Published:** 2017-03-04

**Authors:** Han-Gang Yu, Sarah McLaughlin, Mackenzie Newman, Kathleen Brundage, Amanda Ammer, Karen Martin, James Coad

**Affiliations:** 1 Department of Physiology and Pharmacology, West Virginia University, One Medical Center Drive, Morgantown, WV 26506 USA; 2 Mary Babb Randolph Cancer Center, West Virginia University, Morgantown, WV 26506 USA; 3 Department of Microbiology, Immunology and Cell Biology, West Virginia University, Morgantown, WV 26506 USA; 4 Department of Neurobiology and Anatomy, West Virginia University, Morgantown, WV 26506 USA; 5 Department of Pathology, West Virginia University, Morgantown, WV 26506 USA

**Keywords:** Breast cancer, Triple-negative, Bioelectricity, Calcium channel, Selective killing, Caspase-3

## Abstract

**Background:**

Human triple-negative breast cancer has limited therapeutic choices. Breast tumor cells have depolarized plasma membrane potential. Using this unique electrical property, we aim to develop an effective selective killing of triple-negative breast cancer.

**Methods:**

We used an engineered L-type voltage-gated calcium channel (Cec), activated by membrane depolarization without inactivation, to induce excessive calcium influx in breast tumor cells. Patch clamp and flow cytometry were used in testing the killing selectivity and efficiency of human breast tumor cells in vitro. Bioluminescence and ultrasound imaging were used in studies of human triple-negative breast cancer cell MDA-MB-231 xenograft in mice. Histological staining, immunoblotting and immunohistochemistry were used to investigate mechanism that mediates Cec-induced cell death.

**Results:**

Activating Cec channels expressed in human breast cancer MCF7 cells produced enormous calcium influx at depolarized membrane. Activating the wild-type Cav1.2 channels expressed in MCF7 cells also produced a large calcium influx at depolarized membrane, but this calcium influx was diminished at the sustained membrane depolarization due to channel inactivation. MCF7 cells expressing Cec died when the membrane potential was held at -10 mV for 1 hr, while non-Cec-expressing MCF7 cells were alive. MCF7 cell death was 8-fold higher in Cec-expressing cells than in non-Cec-expressing cells. Direct injection of lentivirus containing Cec into MDA-MB-231 xenograft in mice inhibited tumor growth. Activated caspase-3 protein was detected only in MDA-MB-231 cells expressing Cec, along with a significantly increased expression of activated caspase-3 in xenograft tumor treated with Cec.

**Conclusions:**

We demonstrated a novel strategy to induce constant calcium influx that selectively kills human triple-negative breast tumor cells.

## Background

One of the biggest challenges in breast cancer treatment is the non-selective destruction of healthy cells alongside cancer cells. There are three major clinically identifiable types of breast cancer: estrogen receptor (ER+) and/or progesterone receptor (PR+) positive, human epidermal growth factor receptor 2 amplified (HER2-amplified), and triple-negative (no alterations of ER, PR, and HER2). The intracellular signaling pathways that underlie these subtypes of breast cancer are different and as a result chemotherapeutics that target one pathway will be effective in only some of the breast cancer subtypes [1].

Altered bioelectricity during cell division, embryogenesis, and development has been recognized [2]. In vivo impedance measurement of female breasts with and without tumors revealed lower resistance in malignant tumors than in normal mammary tissues [3]. In excised normal and malignant breast tissues, the conductivity is higher in malignant tissues [4]. In breast tissue cells from patients with infiltrating ductal carcinoma, the resting membrane potential (Em) was found to be −13 mV, independent of estrogen receptor (ER) or progesterone receptor (PR) presence [5]. Normal human breast epithelial cell Em is near -60 mV [5].

Calcium influx has increasingly been recognized to play a critical role in breast cancer [6]. Cell proliferation depends on external Ca^2+^, and a fine-controlled Ca^2+^ influx through voltage-gated calcium channel (VGCC) is a critical signal in cell proliferation [7, 8]. Under physiological conditions, there exists a 20,000 - fold Ca^2+^ ion gradient across plasma membrane ([Ca^2+^]_o_ is close to 2 mM, [Ca^2+^]_i_ is around 100nM) [9]. Thus, calcium influx via VGCC becomes a critical cell signal for a variety of cell functions such as neurotransmission, muscle contraction and cell growth [10].

VGCC have been categorized into Cav1 family (L-type), Cav2 family (N-, P/Q and R-type), and Cav3 family (T-type), based on their biophysical, pharmacological and structural properties [10]. VGCC are found in excitable cells such as neuronal, skeletal muscle and cardiac cells, but are not present in non-excitable healthy human mammary epithelial cells [11]. In primary tumor from patients and breast cancer cell lines Cav3.2 transcripts and protein expression have been detected [12–14]. Cav3 T-type calcium channels have a much faster inactivation compared to Cav1 L-type channels [10]. The brief calcium influx through Cav3.2 T-type channel inactivation in response to depolarization is an essential signal for breast cancer cell growth since blockade of this T-type channel dramatically reduced proliferation of breast cancer cells [14].

The amount of calcium ions entering cells is tightly controlled by voltage-dependent activation and inactivation of VGCC [15, 16]. Disturbance of VGCC gating can significantly change the amount of external calcium ions entering cell. Excessive Ca^2+^ influx has been demonstrated to induce apoptosis and cell death [9, 17, 18]. VGCC are activated by membrane depolarization [10]. However, during sustained membrane depolarization Ca^2+^ entry is limited by channel inactivation, an intrinsic protection mechanism [19, 20]. When the channel inactivation is removed, continuous Ca^2+^ influx leading to excessive accumulation of intracellular Ca^2+^ ions can be generated.

We have recently demonstrated that altering bioelectricity and external calcium concentration can induce death of different types of breast cancer cells, MCF7 and MDA-MB-231 [21]. In the present work, we demonstrated that using an engineered VGCC that lacks inactivation (Cec) [22], massive Ca^2+^ influx can be induced to trigger the death of human carcinoma breast tumor cells, but not in non-tumor human mammary epithelial cells. Cec injected into a human breast tumor in vivo caused cell death within the tumor via caspase-3 and inhibited tumor growth. This approach of selective killing of human breast cancer cells is independent of ER+/PR+, HER2 and triple-negative subtypes.

## Methods

Human mammary non-tumor and cancer cell lines were purchased from American Tissue Culture Center (ATCC) (non-tumor cells MCF10A, CRL-10317; MCF7 cell line, HTB-22; MDA-MB-231 cell line: HTB-26).

### Cell culture and plasmid transfection

Human breast adenocarcinoma cells (ER-positive MCF7, triple-negative MDA-MB-231/Luc) and human mammary gland epithelial cells (MCF10A) were grown in Dulbecco’s modified Eagle’s medium (DMEM, Invitrogen), supplemented with 10% fetal bovine serum, 100 IU/mL penicillin, and 100 g/L streptomycin. Normal human mammary epithelial cells (HMEC) were grown in mammary epithelial cell basal medium (MEC), supplemented with MEC growth kit (ATCC). For patch clamp studies, HMEC and MCF7 cells were grown on poly-D-lysine coated coverslips in DMEM and MEC, respectively.

Cells with 50–70% confluence in 6-well plates were used for transient plasmid transfection (1–2 μg for each plasmid). We used three methods for plasmid transfection: Lipofectamine2000 transfection reagent (Invitrogen), MCF7 transfection reagent (Altogen) and Nucleofection (electroporation, Lonza).

Cav1.2 and Cec, both in pCEP4 mammalian expression vector, were either co-transfected with GFP or fused with mCherry for verification of expression and served as selection guidance for patch clamp studies and flow cytometry experiments. The beta subunit of Cav1.2, β2a, was co-transfected with Cav1.2 and Cec to enhance the surface expression of the channel proteins [16].

### Patch clamp studies

From day 1 up to day 4 post-transfected cells with green fluorescence were selected for patch clamp studies. The cells grown on coverslips were placed in a lucite bath with the temperature maintained at 35–37 °C. Em and voltage-gated calcium currents were recorded using the whole cell patch clamp technique with an Axopatch-700B amplifier. Em was measured with normal Tyrode and pipette solutions. The Tyrode solution contained (in mM): 143 NaCl, 5.4 KCl, 1.8 CaCl_2_, 0.5 MgCl_2_, 0.25 NaH_2_PO_4_, and 5 HEPES; pH was adjusted to 7.4 by NaOH. The pipette solution contained (in mM): 120 KCl, 1 CaCl_2_, 5 MgCl_2_, 5 Na2ATP, 11 EGTA, 10 HEPES, and 11 glucose; pH was adjusted to 7.3 by KOH.

For I_CaL_ recordings, the pipettes had a resistance of 2–5 MΩ when filled with (in mM): 108 CsCl, 4 MgCl_2_, 9 EGTA, and 9 HEPES. The bath solution contained (in mM): 2 CaCl_2_, 1 MgCl_2_, 10 HEPES, 10 TEA, 10 glucose, and 100 CsCl. Both pipette and bath solutions were adjusted by CsOH to pH 7.2. P/4 protocols were used to remove the leak currents [23]. The whole-cell patch clamp data were acquired by CLAMPEX and analyzed by CLAMPFIT (pClamp 9, Molecular Device/Axon).

### Live cell imaging

Live cell imaging experiments were performed using a Zeiss Axio Observer A1 inverted microscope with fluorescence. Images were acquired and analyzed using AxioVision (version 4.6). Live cell imaging with patch clamp experiments were performed using Slidebook (version 4.0). We used ethidium homodimer-1 (EthD-1, Invitrogen) dye (0.2–0.5 μl of 2 mM stock to 1 ml culture of cells in 6-well plate) to label dead cells. EthD-1 dye enters the cell only after the plasma membrane is disintegrated, it then binds to DNA in the nucleus and emits red fluorescence (Invitrogen) [24].

### Flow cytometry

Cell death was examined using a Live/Dead Fixable Violet Viability kit (Life Technologies) following the manufacturer’s instructions. Briefly, cells were transfected and cultured as described above. After trypsinization and washing, cell pellets were resuspended in PBS and stained with the Live/Dead dye in the kit. Samples were then analyzed on a BD LSRFortessa analytical flow cytometer (BD Biosciences, San Jose CA) using BD Diva 8.0 software. The GFP signal was detected using a 488 nm sapphire laser and a 530/30 BP filter in front of the detector. The Violet live/dead signal was detected using a 405 nm OBIS laser and a 450/50 BP filter in front of the detector. Between 25,000 and 30,000 events were collected for each sample.

### Lentivirus preparation

Cec was inserted into a GFP tagged lentiviral vector, pCDH-CMV-MCS-EF1-GFP + Puro (System Biosciences, SBI) between SwaI and NotI in MCS to yield lenti-GFP-Cec plasmid. The empty lentiviral vector fused with GFP (lenti-GFP) was used as a negative control. Lentivirus was produced using pPACK lentivector packaging systems following the vendor’s instruction manual (SBI). The concentration of lentivirus was 1-2×10^12^ LP/ml (LP: lentiviral particle). For treatment groups, 5-10ul of lenti-GFP-Cec was injected directly into the primary tumor. For control groups, 5–10ul of lenti-GFP was injected directly into the primary tumor. The same amount of virus was administrated to each group per experiment.

### Breast tumor induction in NOD scid gamma (NSG) mice

Female NSG-immunodeficient mice were purchased from the Jackson Laboratory. Animals were housed in the WVU Animal Facility under protocols approved by the Institutional Animal Care and Use Committee. For mammary pad injections, pathogen-free luciferase-expressing human breast adenocarcinoma cells (MDA-MB-231/Luc, 1-2×10^6^ cells/animal) were injected into the fourth inguinal mammary gland of 6- to 8-week-old mice. Primary tumors had formed one week of injection.

### Primary tumor imaging

Animals were anesthetized by exposure to 1–3% isoflurane during imaging. Imaging was performed weekly over the course of each experiment, typically for 4–6 weeks.

#### Tumor size measurement by bioluminescence imaging (BLI)

D-Luciferin (150 mg/kg) was injected into the peritoneum. Images were obtained after ten minutes’ exposure using an IVIS Lumina-II Imaging System and Living Image-4.0 software (PerkinElmer). Luciferin-sensitive bioluminescence signals (photons/s) were used to assess the tumor size.

#### Tumor volume measurement by ultrasound imaging (USI)

Tumor volume was imaged by Vevo2100 Micro-Ultrasound System. A 40 or 50 mHz transducer was used, depending on the tumor volume. A 3-dimensional (3D) image was acquired with scanning distance of 0.071 mm between images. Vevo software then integrated the images into a reconstructed 3D tumor from which the tumor volume was obtained.

#### Tumor fluorescence imaging

Tumor imaging was performed with an Olympus MVX10 MacroView microscope in conjunction with cellSens software version 1.11. Images were acquired using a 1X objective at 0.8X zoom. Bright field images were rendered at around 20 ms exposure in 16-bit grayscale, while fluorescence was captured at around 700 ms in 8-bit RGB mode. Image overlays were converted to 8-bit RGB color before analysis.

### Histology of NSG tumors

Primary tumors were removed from NSG mice, fixed in 4% (m/v) formaldehyde for 16 hrs and embedded in paraffin. Paraffin blocks were cut to 5-μm-thick sections for hematoxylin and eosin (H&E) stain. Imaging of H&E slides was performed with an Olympus MVX10 MacroView microscope equipped with dual cameras, Hamamatsu (fluorescence imaging) and DP26 (color imaging). Necrotic area was quantified using ImageJ (NIH).

### Western blotting

MDA-MB-231 cells were and transfected with either a lentiviral GFP construct, wild-type Cav1.2, Cec, or empty vector as a control. After three days of incubation, adherent cells were lysed in buffer (20 mM Tris, 150 mM NaCl, 10 mM EGTA, and 10 mM EDTA at pH ~ 7.4) with a cell scraper and allowed to sit for five minutes. Detached cells were pelleted and resuspended in the lysis solution. After centrifugation at 3,000xg for five minutes, supernatant protein concentration was measured using Bradford's method.

SDS-PAGE was carried out in 4–12% gradient bis-tris gels (Invitrogen) on 10–20 μg of total protein. Samples were then transferred to 0.2 μm pore PVDF membranes (Thermo Scientific). After blocking with 3% BSA-V (Sigma) in TBST (Invitrogen), caspase-3 antibody (Abcam) was added in a 1:1000 dilution and left to incubate overnight at 4 °C, followed by incubation with secondary antibody in a 1:5000 dilution. After one hour, membranes were washed five times for five minutes in TBST and visualized using a standard ECL kit (Life Technologies).

### Immunohistochemistry of NSG tumors

Immunohistochemistry (IHC) detection of cleaved caspase-3 in formalin-fixed paraffin-embedded (FFPE) tumor tissue was performed according to vendor’s manual instruction (Biocare) and following a verified protocol in the Pathology Laboratory of Translational Medicine at WVU. Briefly, 3 μm sections were deparaffinized on slides, quenched with hydrogen peroxide, and incubated in cleaved caspase-3 antibody (prediluted rabbit polyclonal antibody, Biocare Medical, Concord, CA, 1:200 dilution) at 4 °C for 4 min. Horseradish peroxidase-containing secondary antibody (UMap anti-RB, Roche, Diagnostic, Cupertino, CA) was then added for 8 min and developed using Biocare DAB (brown color). Hematoxylin was used as a counterstain (blue color).

Caspase-3 IHC slides were examined under an Olympus AX70 Provis microscope equipped with an Optronics MicroFire color CCD camera and a 20x/0.70 UPlanApo objective or a 40x/0.90 UApo objective. Images were acquired using Stereo Investigator 10 (MDF Bioscience). Quantification of cells expressing cleaved caspase-3 was performed by ImageJ [25].

### Statistical analysis

Data were shown as mean ± standard deviation (SD) in the text. Bar figures were presented as mean ± SD using GraphPad 4 (Prism). Student’s *t*-test and two-way ANOVA (for more than two groups) were used for statistical analysis. *P* < 0.05 was considered as statistically significant.

## Results

### Voltage-gated calcium currents in normal and tumor mammary epithelial cells

**Fig. 1:**
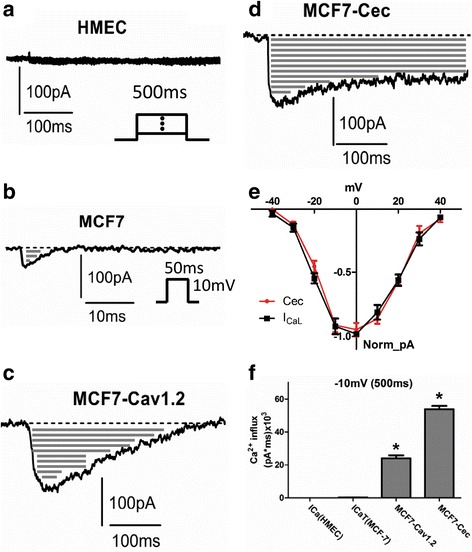
Ca^2+^ influx through L-type voltage-gated calcium channels in breast cancer cells. **a** Lack of voltage-gated calcium currents in response to the 500 ms depolarizing pulses from -50 mV to +50 mV in 10mv increments (inset). **b** Representative T-type calcium current in a MCF7 cell (inset is the pulse protocol). **c** After 3-day transfection of Cav1.2 in MCF7 cells, L-type calcium current was elicited by a 500 ms depolarizing pulse to -10 mV; gray lines show the Ca^2+^ influx. **d** After 1-day transfection of Cec, L-type calcium current was elicited by a 500 ms depolarizing pulse to -10 mV. *Gray lines* in the enclosed area in **b**, **c**, and **d** indicate the Ca^2+^ influx. **e** Normalized current–voltage (IV) relationship of L-type Cav1.2 (I_CaL_) and Cec expressed in MCF7 cells (*n* = 6 in each group), the membrane potential was held at -80 mV. **f** Ca^2+^ influx generated by T-type calcium current, L-type calcium current, and Cec calcium current in response to a 500 ms depolarizing pulse to -10 mV, respectively (*n* = 6). * indicates statistical significance compare to I_Ca_ in MCF7 and in HMEC groups. The membrane potential was held at -80 mV

Patch clamp studies confirmed absence of voltage-gated calcium currents in normal HMEC cells (Fig. 1a) and only T-type voltage-gated calcium current (I_CaT_) in MCF7 cells (Fig. 1b). T-type calcium current was verified by its fast activation and inactivation as well as blockage by 1 mM Mn^2+^ and 0.1 mM Ni^2+^ ions [10, 26] (dotted line in Fig. 1b).

### Properties of L-type VGCC and engineered channel (Cec) expressed in MCF7 cells

L-type calcium channels are activated at a depolarized Em, around -40 mV associated with 10–100 times slower inactivation compared to T-type calcium channel [10]. Cav1.2 L-type calcium channel was co-transfected with GFP into MCF7 cells. L-type calcium current (I_CaL_, I_Cav1.2_) was elicited by a 500 ms depolarizing pulse to -10 mV (Fig. 1c). Inactivation of I_CaL_ is significantly slower compared to that of I_CaT_ (or I_Cav3.1_) (Fig. 1b) (note the different time scales used in 1b and 1c). L-type calcium current was verified by a specific L-type calcium channel blocker, verapamil (10 μM) (dotted line in Fig. 1c).

Cec, an engineered Cav1.2 channel with defective inactivation, was expressed in MCF7 cells. Cec current (I_Cec_) was activated by a 500 ms depolarizing pulse to -10 mV (Fig. 1d). Cec has the same activation as Cav1.2 but lacks inactivation, i.e. the current trace does not come back to the baseline, as T-type and wild-type L-type calcium currents do. Cec channel has a similar current–voltage relationship as Cav1.2 (Fig. 1e).

Calcium influx through voltage-gated calcium channels is indicated by the gray lines enclosed by the current trace and the dotted baseline (Fig. 1b, c, d). In response to a 500 ms depolarizing pulse to -10 mV from a holding potential of -80 mV, Cec induced more than a 50,000-fold larger amount of calcium influx than was induced by endogenous T-type channels in MCF7 cells (Ca-flux_HMEC: 0.001 ± 0.001 pA*ms; CaT-flux_MCF7: 0.24 ± 0.15 pA*ms; Cav1.2-flux_MCF7: 24.0 ± 3.8 pA*ms; Cec-flux_MCF7: 53.8 ± 5.1 pA*ms; Fig. 1f). Under the same pulse protocol, Cav1.2 induced more than a 20,000-fold higher calcium influx compared to T-type calcium channel (Fig. 1f).

### Cec-induced cell death in MCF7 cells

**Fig. 2:**
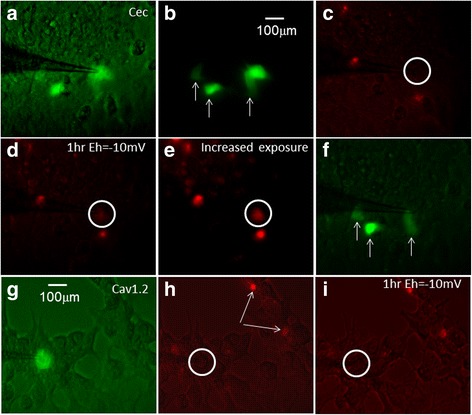
Depolarization-triggered cell death in a single Cec-expressing MCF7 cell. **a** After 1 day transfection a Cec-expressing cell was clamped by a patch pipette. **b** A focused region of four Cec-expressing cells showing green fluorescence. **c** Ethd-1 was used to stain dead cells. Note that the patched cell in a *white circle* was live (no *red color*) prior to membrane depolarization. **d** After 1 h of depolarizing membrane to -10 mV, the patched cell was dead shown by the red color in the *white circle*. **e** Enhanced exposure to illustrate the red color in the white circle. **f** In comparison to (**b**) the dead cell was confirmed by loss of green fluorescent signal while other 3 Cec-expressing cells not subjected to membrane depolarization were still alive. **g** A Cav1.2-expressing MCF cell patched by a patch pipette, **h** Ethd-1 demonstrated that the patched cell was alive (in the *white circle*), the *white arrows* indicated dead cells used as a reference. **i** After membrane depolarization to -10 mV for 1 h, the cell was still alive without the *red color*. *White circle* in H and I indicates the patched cell. Similar results were obtained in an additional 5 cells

A dramatic increase in calcium influx is lethal to cell survival. To test whether excessive calcium influx through Cec channels can indeed trigger cell death, we activated Cec while simultaneously monitoring the cell’s status using fluorescence imaging. Figure 2a shows a Cec-expressing MCF7 cell clamped by a patch pipette. Figure 2b shows three neighboring Cec-expressing cells that were not patch clamped. Figure 2c shows the naturally dead cells stained by Ethd-1 (red color), which contrast the patched live cell shown in the white circle. After holding the cell membrane at–10 mV for 1 h, the patched cell was dead as indicated by the appearance of red color (Fig. 2d). Figure 2e shows the same image with increased exposure time for clarity. Death of the patched cell was confirmed by disappearance of GFP and the survival of its neighboring cells which also expressed Cec but were not voltage-clamped at–10 mV (Fig. 2f versus Fig. 2b). Cells co-expressing Cav1.2 and GFP under the same experimental conditions showed no sign of dying (Figs. 2g–i).

**Fig. 3:**
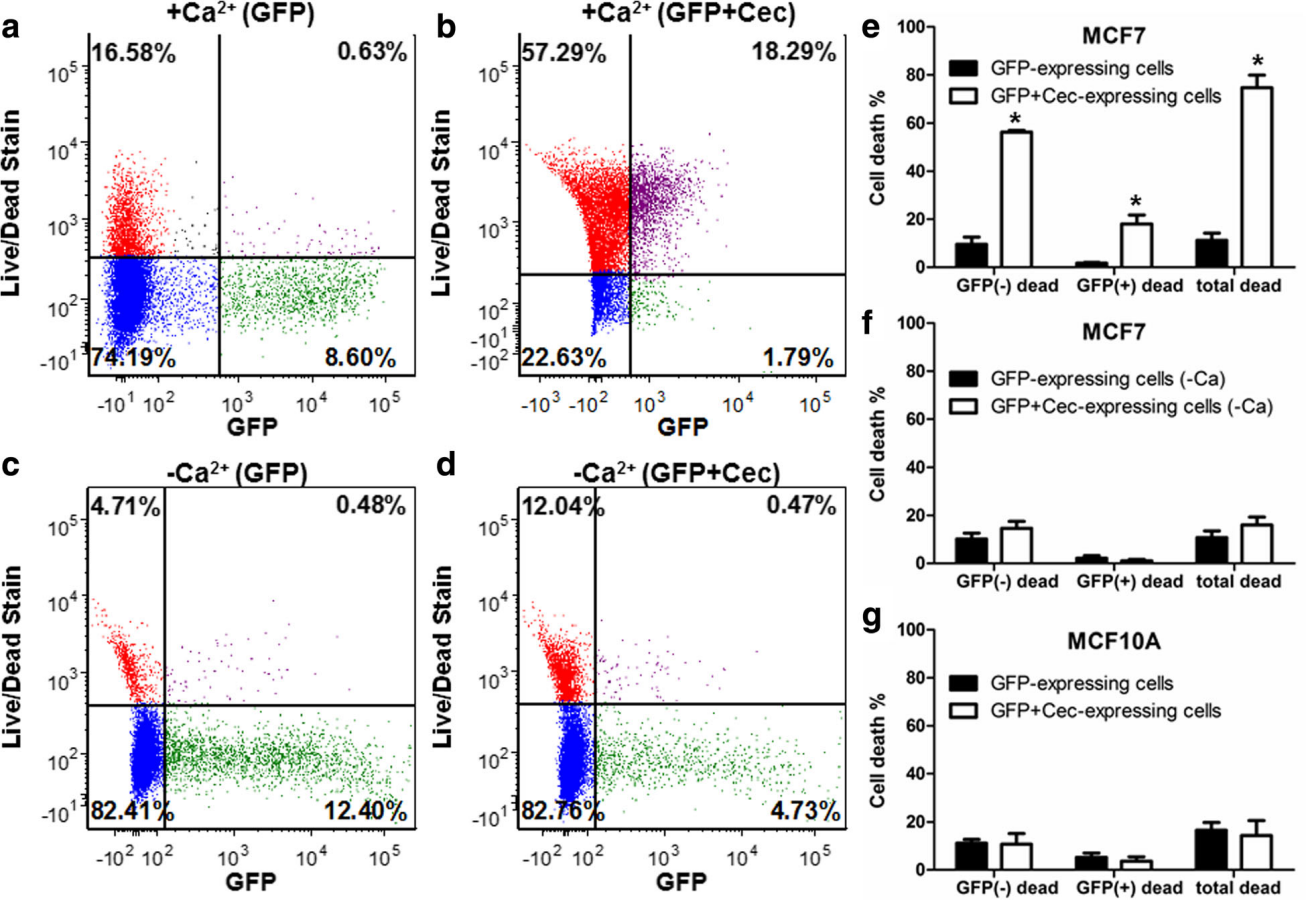
Cec-induced cell death from flow cytometry. **a** MCF7 cells transfected with only GFP in DMEM containing 1.8 mM Ca^2+^. **b** MCF7 cells co-transfected with GFP and Cec in DMEM containing 1.8 mM Ca^2+^. **c** MCF7 cells transfected with only GFP in DMEM without Ca^2+^. **d** MCF7 cells co-transfected with GFP and Cec in DMEM without Ca^2+^. **e** Percentage of dead cells between GFP-expressing and Cec-expressing MCF7 groups in the presence of Ca^2+^, * indicates *p* < 0.001 in all three groups (*n* = 6). **f** Percentage of dead cells between GFP-expressing and Cec-expressing MCF7 groups in the absence of Ca^2+^, *p* > 0.05 in all three groups (*n* = 5). **g** Percentage of dead cells between GFP-expressing and Cec-expressing MCF10A cells, *p* > 0.05 in all three groups (*n* = 5)

To examine the efficacy of Cec-induced cell death, we compared the percent of cell death in GFP-expressing or Cec-expressing MCF7 cells using flow cytometry. After 3 days of transfection, Cec induced significantly more cell death (Fig. 3b) than GFP alone (Fig. 3a) in both GFP(−) and GFP(+) populations (upper left and upper right quadrants). In the absence of Ca^2+^ ions, the flow cytometry results were similar in Cec-expressing and non-Cec-expressing cells (Fig. 3c, d). On an average, there was a 9-fold increase in GFP(+) dying cells in Cec-expressing than in GFP-expressing MCF7 cells (Fig. 3e) (GFP(+) dead were 18.3 ± 8.6% in Cec-expressing cells and 1.7 ± 0.7% in GFP-expressing cells, respectively, *n* = 6). In addition, GFP(−) cell death was increased by 8-fold in Cec-expressing (58.8 ± 10.5%) than in GFP-expressing (6.9 ± 6.6%) MCF7 cells (*n* = 6). It should be noted that GFP(−) dead cells included both untransfected cells and Cec-expressing cells that have degraded GFP protein.

When calcium was removed from the culture medium, there was no statistically significant difference in percentage of cell death between GFP-expressing and Cec-expressing groups (Fig. 3f) (GFP(+) dead were 1.2 ± 1.2% in Cec-expressing cells and 2.2 ± 2.7% in GFP-expressing cells, *n* = 6). The percentage of cell death in GFP(−) dead in Cec-expressing cells was also significantly decreased when Ca^2+^ was absent (74.7 ± 12.7% in the presence of Ca^2+^ and 16.1 ± 7.8% in the absence of Ca^2+^, *n* = 6).

MCF10A (a non-tumorigenic human breast epithelial cell line) cells have been commonly used as a control to MCF7 cells [27]. We found the resting membrane potential of MCF10A to be −53.5 ± 8.2 mV (*n* = 4), in agreement with a previous report [5]. Flow cytometry results showed that in Ca^2+^ - containing culture medium, there is no statistically significant difference between GFP-expressing cells and Cec + GFP expressing cells (Fig. 3g). (GFP(+) dead were 3.7 ± 3.1% in Cec-expressing cells and 5.3 ± 3.0% in GFP-expressing cells, *n* = 3).

### Cec-induced inhibition of tumor growth in NSG mice

**Fig. 4:**
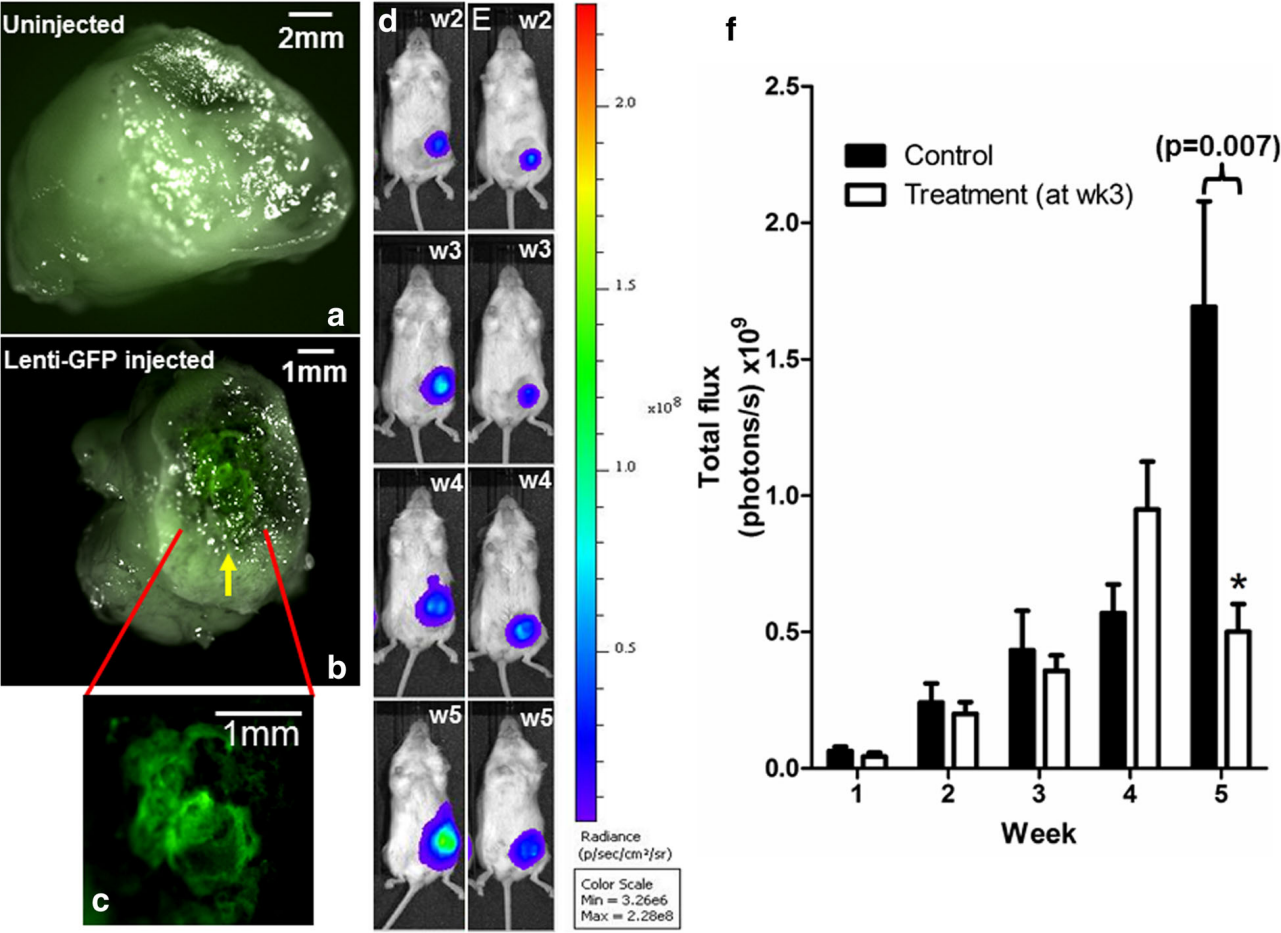
Fluorescence imaging of primary tumors, tumor size assessment by bioluminescence imaging. **a** Primary tumor without virus injection. **b** Primary tumor injected with lenti-GFP control virus. Yellow arrow indicates the GFP signal. **c** Enlarged GFP fluorescence from B. The control group (dark bar) was injected with lenti-GFP and the treatment group (white bar) was injected with lenti-GFP-Cec. The lentivirus injection was performed at week 3. BLI was taken weekly for the control mouse (**d**) and the treated mouse (**e**) from week 2 to week 5. **f** BLI signals, total flux (photons per second), were shown for control group (*n* = 3) and for treatment group (*n* = 5). * indicates significant difference compared to the control group at week 5

To further explore whether Cec-mediated Ca^2+^ influx can inhibit breast tumor growth in vivo, we used a commonly used human breast tumor mouse model, NOD scid gamma (NSG) mice. The xenograft tumors in NSG were induced by injection of MDA-MB-231/Luc cells. MDA-MB-231 is a human breast triple-negative cancer cell line [28]. The resting membrane potential was measured to be −39.48 ± 12.14 (*n* = 7), similar to MCF7. After three weeks of tumor growth, lentivirus injection was performed. Control mice were injected with lenti-GFP and treatment mice were injected with lenti-GFP-Cec directly into the tumor. To confirm the presence of lentivirus within the tumor after injection, the primary tumor was removed and subjected to fluorescence imaging. Figure 4a shows the absence of GFP fluorescence within a tumor which received no injection of lentivirus. No GFP fluorescence was observed in tumors injected by lenti-GFP-Cec, presumably due to the cell death. Tumors injected with lenti-GFP showed GFP fluorescence (Fig. 4b, white arrow; Fig. 4c – enlarged GFP image). Similar observation was seen in at least three control mice.

Tumor size was measured by bioluminescence imaging (BLI) illustrated in 4D (control mouse) and 4E (treatment mouse). Changes BLI for control and treatment groups are shown in Fig. 4f. Lentivirus injection was performed in week 3. There are no statistically significant changes in BLI between the two groups until week 5. At week 5, the tumor size was 70% smaller in treatment group (0.5 ± 0.2 (photons/s) ×10^9^, *n* = 6) compared to control group (1.69 ± 0.79 (photons/s) ×10^9^, *n* = 4, *p* = 0.007). In treatment group, BLI was also decreased by 53% at week 5 (0.5 ± 0.2 (photons/s) ×10^9^) compared to week 4 (1.06 ± 0.32 (photons/s) ×10^9^, *p* = 0.015).

**Fig. 5:**
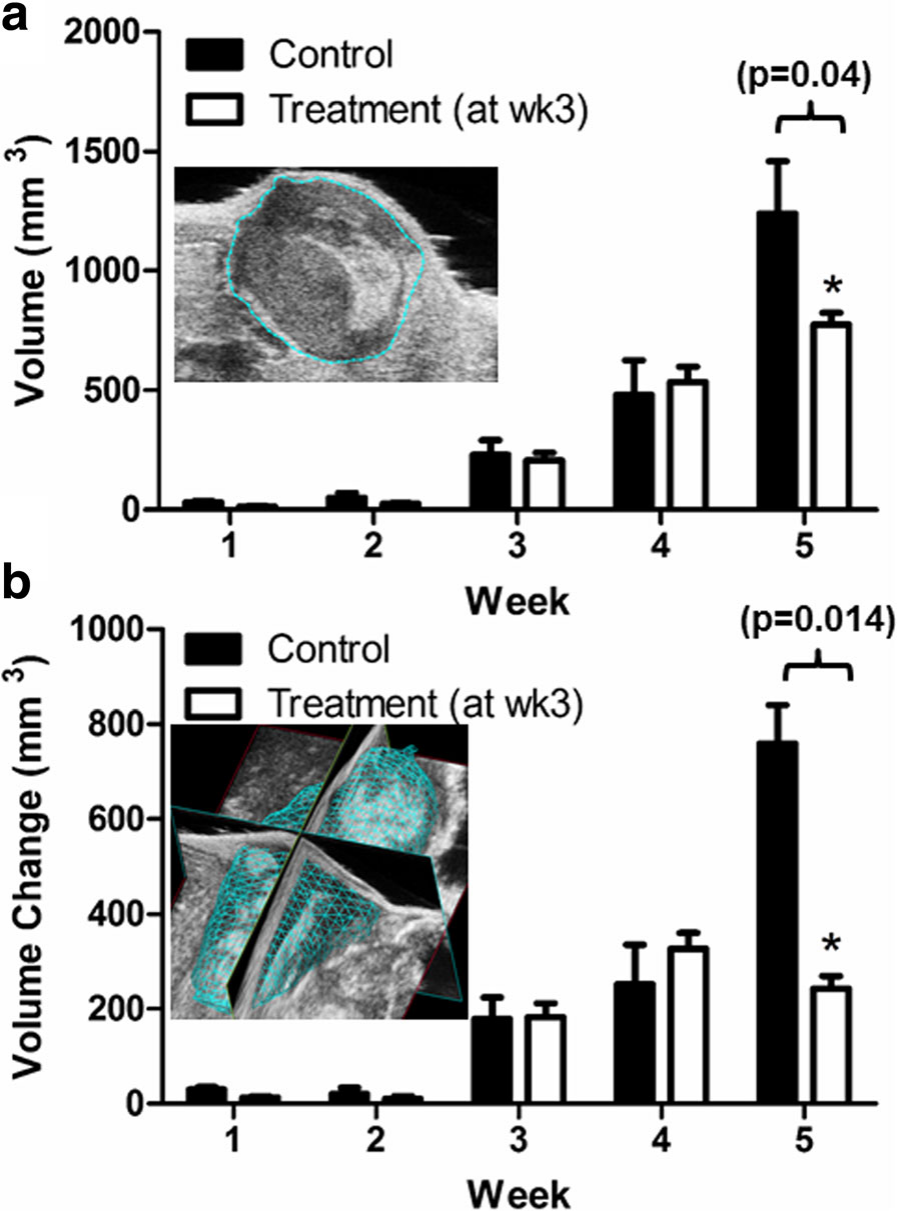
Tumor volume weekly measured by ultrasound imaging. The lentivirus injection was performed at week 3. The control group (*dark bar*) was injected with lenti-GFP and the treatment group (*white bar*) was injected with lenti-GFP-Cec. Tumor volume is shown in (**a**) and tumor volume change is shown in (**b**). The inset in (**a**) shows the drawing contour of the tumor, the inset in (**b**) shows the 3D reconstitution of the tumor. The volume change was calculated by dV(n) = Vn – V (n-1), where *n* = 1 to 5. Data were averaged from 3 control mice and 5 treated mice. * indicates significant difference compared to the control group at week 5

Figure 5 shows the tumor volume measured by ultrasound imaging (USI). For the first four weeks there are no statistical differences in tumor volumes between the control and treatment groups (*p* > 0.05). At week 5, tumor volume was 37% smaller in the treatment group than in the control group (control: 1240 ± 379 mm^3^, *n* = 3, treatment: 776 ± 112 mm^3^, *n* = 5, *p* < 0.05) (5a). From week 4 to week 5, the tumor grew by 158% in control group (week 4: 480 ± 251 mm^3^, week 5: 1240 ± 379 mm^3^, *n* = 3, *p* < 0.05), whereas the tumors grew 46% in the treatment group (week 4: 533 ± 146 mm^3^, week 5: 776 ± 111 mm^3^, *n* = 5, *p* < 0.05) (5a). Over 3-fold slower growth in treatment group was due to the smaller change in volume compared to control group from the previous week (for difference between week 4 and week 5, control = 760 mm^3^; treatment = 243 mm^3^) (5b).

### Cec-induced cell death within MDA-MB-231 xenograft

Hematoxylin & eosin (H&E) staining was performed for Cec-treated (6a) and control (6b) tumors. Close to 23% of area within the control tumors was dead (22.6 ± 15.2%, *n* = 4) and nearly 76% of area within the treated tumors was dead (75.7 ± 5.3%, *n* = 4) (6c).

Concerning naturally occurred necrosis in large tumors (defined as the volume greater than 200 mm^3^, length greater than 7 mm), we also examined dead or dying cells in small tumors (defined as the volume less than 200 mm^3^, length less than 7 mm). Nitro blue tetrazolium (NBT) staining indicated that dead/dying cells were visible in a typical small tumor injected with Cec-containing lentivirus (6d, dash yellow line), but barely detected in the small tumor injected with control lentivirus (6e). Although the average tumor volume was similar between the two groups (V_treat = 164.1 ± 90.0 mm^3^, V_control = 132.9 ± 131.6 mm^3^, *n* = 3, *p* > 0.05), the percent growth of tumor is 7-fold smaller in treat group (26.7 ± 23.9%) than in control group (201.5 ± 123.8%) (6 F, *n* = 3, *p* < 0.05).

Excessive Ca^2+^ influx induced by Cec can trigger apoptosis commonly mediated by caspase activation [9]. Among several caspases present in the MDA-MB-231 cells, caspase-3 is the most commonly activated one in response to a variety of anti-cancer drugs that induce cell death [29–31]. The active caspase-3 protein expression was detected only in MDA-MB-231 cells expressing Cec, but absent in cells expressing control vector (GFP) and untransfected (N) cells (6 g). In small MDA-MB-231 xenograft tumors, the cleaved (active) caspase-3 expressing cells was barely detected (5.4 ± 3.0% in untreated and 14.3 ± 4.0% GFP treated group, respectively, *n* = 4) (6 h, j), but significantly expressed in Cec-treated group (50.4 ± 19.0%, *n* = 4) (6i, j).

## Discussion

Excessive negative charges at the external surface of plasma membranes due to lower K^+^ concentration and higher Na^+^ concentration in cancer cells was observed nearly 50 years ago [32, 33]. As a result, the resting plasma membrane potential is depolarized in cancer cells. The intrinsic link between membrane depolarization and tumorigenesis has been elegantly illustrated by mitosis in mature neurons induced by sustained depolarization [34].

Depolarization can activate a variety of ion channels that elicit multiple cell functions. One of these ion channels is a voltage-gated calcium channel [20]. Ca^2+^ influx following the opening of VGCC is a critical signal for cell proliferation [9]. Recently, membrane depolarization has been recognized as an early bioelectric signal which can serve as a diagnostic marker for cancer development, as hyperpolarization was shown to inhibit tumor formation [35].

We found a significant membrane depolarization in MCF7 and MDA-MB-231 cells compared to normal HMEC, in agreement with previous observations both in cell lines and primary tumors [5]. It is noted that use of whole-cell patch clamp technique offers advantages over microelectrode measurement of tissue for accurate measurements of plasma membrane potential [36]. We also found no voltage-gated calcium currents in normal mammary epithelial cells, but T-type calcium currents in MCF7 cells with a typical voltage-dependent activation from -70 mV to +40 mV (Figs. 1c, d). These results support the notion that T-type calcium current serves as a functional biomarker in human breast cancer cells.

To test our hypothesis for selectively killing of breast cancer cells by inducing excessive Ca^2+^ influx, we chose Cav1.2 because it is not present in either HMEC or MCF7 cells [11, 37] (Fig. 1b). Cav1.2 began to activate at a membrane potential more positive than -40 mV, +30 mV more positive than T-type calcium current activation threshold. Thus, introducing Cav1.2 into cells should not alter cell function unless the cell is depolarized to -40 mV.

In comparison to Cav1.2, Cec has a similar activation threshold (Fig. 1h) but the inactivation current trace was unable to return to baseline (Fig. 1g), leading to defective closure of the channel. As a result, external calcium ions can constantly enter the cell. For a 500 ms depolarizing pulse, opening of Cec channels allowed more than a 50,000-fold higher amount of calcium influx compared to T-type calcium channels (Fig. 1j). We noted that Cav1.2 channels can also induce more than a 20,000-fold higher amount of calcium influx over T-type calcium channels (Fig. 1j). The difference between Cav1.2 and Cec, however, is that Cav1.2 channels can close (becoming inactivated) during sustained membrane depolarization, while Cec cannot.

At single-cell level, we showed that the Cec-expressing cells died when the membrane was held at -10 mV for an hour (Fig. 2d–f). Neighboring Cec-expressing cells that were not depolarized to -10 mV did not die (Fig. 2b, f). This experiment demonstrated the selectivity of Cec-mediated killing of cancer cells. It also revealed difficulty in killing cancer cells expressing the Cav1.2 channel (Fig. 2g–i). Although opening of Cav1.2 can induce more than a 20,000-fold amount of Ca^2+^ influx compared to non-Cav1.2 expressing cells, the cells did not die due to inactivation of the channel.

The killing of MCF7 cells via a Cec-induced increase in Ca^2+^ influx was further tested in cell monolayer. Flow cytometry data (Fig. 3) confirmed that Cec activation can indeed kill MCF7 cells but not MCF10A cells and that Ca^2+^ is the critical factor in Cec-mediated cell death.

**Fig. 6:**
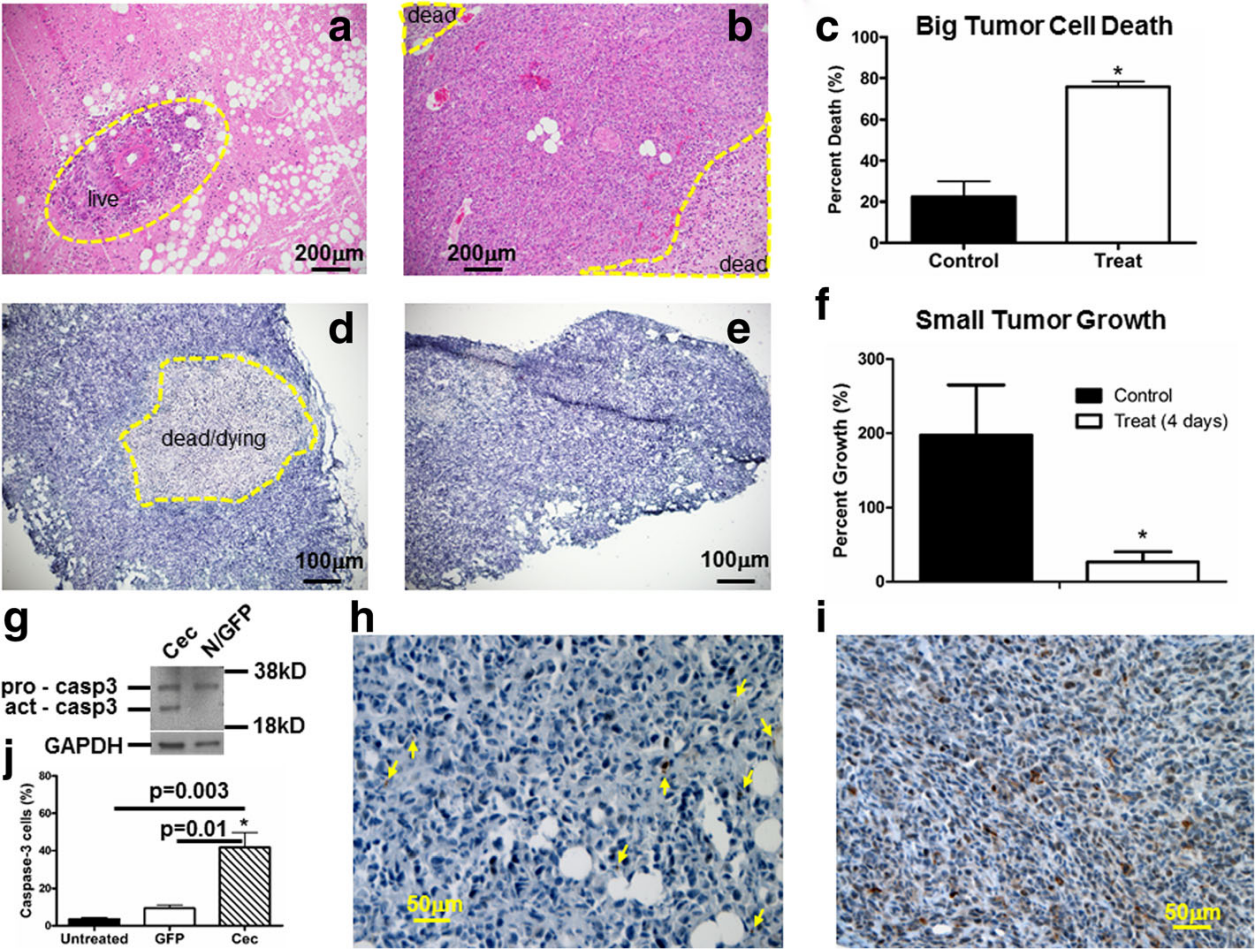
Cec-induced cell death in NSG tumors. Tumors at week 6 were removed. H&E staining was performed in a treated tumor (**a**) and a control tumor (**b**). **c** Percentage of dead cells in control and treat mice. Tumors at week 3.5 were removed. Live/dead NBT (nitro blue tetrazolium) staining was performed in a treated tumor (**d**) and in a control tumor (**e**). Yellow dash line indicates live (**a**) or dead cells (**b**, **d**). **f** Percent growth of treated and control tumors. * indicates statistical significance in comparison to the control tumor. **g** The pro-caspase was detected in MDA-MB-231 cells near 36 kDa (N for non-transfection). The active caspase appeared around 20 kDa in cells expressing Cec but not GFP (another negative control) or non-transfection (N). GAPDH was used as the loading controls. Cleaved caspase-3 expression (cells in brown color) is shown in untreated small tumor (yellow arrows) (**h**) and in Cec-treated small tumor (**i**), respectively. **j** Percentage of caspase-3 expression cells in untreated or treated with empty lentiviral vector and Cec-treated small tumors. * indicates statistical significance comparing to untreated or GFP treated tumor

To verify whether the results obtained in vitro work in vivo, we injected 1-2 × 10^6^ MDA-MB-231/Luc cells into 6–8 weeks old female NSG mice. Primary tumor can grow to 0.1–0.3 mm^3^ volumes after 2–3 weeks, which allows direct injection of virus into the primary tumor (Fig. 3a-c). The tumor volumes were assessed and measured by bioluminescence and ultrasound imaging, respectively. There is a noticeable difference in tumor measurements using BLI and USI. Two weeks after virus injection (at week 3), BLI showed a decrease in tumor size (Fig. 4f), whereas USI showed continuing growth of the tumors (although at a slower rate) (Fig. 5b). This difference may be due to two different imaging mechanisms. BLI signals depend on luciferin binding to luciferase in MDA-MB-231 tumor cells. Dead or dying cells cannot yield BLI signals due to disrupted luciferase structure, but they contribute to the aggregate anatomical structure captured by USI. Nevertheless, both BLI and USI measurements demonstrated consistent results: comparing to the control mice, tumor growth in the treatment group was significantly inhibited by Cec. This conclusion is further confirmed by histological examination demonstrating that the Cec-induced inhibition of tumor growth was a direct consequence of markedly increased cells death within the tumor (Fig. 6).

Apoptosis is most commonly mediated by caspase activation [9]. MDA-MB-231 cells have at least four isoforms of caspases (caspase-3, −6, −8, −9) [31]. We found that the activated caspase-3 expression was detected only cells expressing Cec (Fig. 6g), in agreement with a high prevalence of cleaved caspase-3 in MDA-MB-231 xenograft tumors treated by Cec (Figs. 6i, j). Thus, the Cec-induced cell death is at least partially medicated by activation of caspase-3.

### Limitations of this research in clinical perspective

This work was focused on proof-of-concept of finding a way to treat breast cancer independent of subtypes. It is imperative to point out the limitations of this research in clinical implication. Calcium influx is a vital signal in many physiological activities such as neuronal action potential, heartbeat, and muscle contraction. Disruption of delicate intracellular calcium concentration can cause mental, cardiac, and muscle disorders. Thus, this research can only be applied locally in mammary tissue to avoid global adverse effects. Additionally, efficiency of introducing Cec into mammary epithelial cells in vivo has yet to be tested, although in cell culture lentiviral particles that contain Cec can achieve over 95% transfection rate. These limitations are the driving forces in future direction of this research.

## Conclusions

In conclusion, we presented data that supports a new strategy for selectively triggering tumor cell death: excessive Ca^2+^ influx induced only in the depolarized plasma membranes of breast cancer cells. This strategy is independent of subtypes of breast cancer and may have a therapeutic application in triple-negative breast cancer that currently lacks targeted therapies.

## Abbreviations

BLI: Bioluminescent imaging
Cec: Engineered voltage-gated L-type calcium channel
Em: Resting membrane potential
HMEC: Human mammary epithelial cell
IHC: Immunohistochemistry
MCF10A: Human non-tumor mammary epithelial cell
MCF7: Human breast cancer cell line
MDA-MB-231: Human triple-negative breast cancer cell line
USI: Ultrasound imaging
WB: Western blot
